# A Novel High Throughput Invasion Screen Identifies Host Actin Regulators Required for Efficient Cell Entry by *Toxoplasma gondii*

**DOI:** 10.1371/journal.pone.0064693

**Published:** 2013-05-31

**Authors:** Rajshekhar Y. Gaji, My-Hang Huynh, Vern B. Carruthers

**Affiliations:** Department of Microbiology and Immunology, University of Michigan Medical School, Ann Arbor, Michigan, United States of America; Seattle Biomedical Research Institute, University of Washington, United States of America

## Abstract

*Toxoplasma gondii* critically relies on cell invasion as a survival strategy to evade immune clearance during infection. Although it was widely thought that *Toxoplasma* entry is parasite directed and that the host cell is largely a passive victim, recent studies have suggested that host components such as microfilaments and microtubules indeed contribute to entry. Hence to identify additional host factors, we performed a high-throughput siRNA screen of a human siRNA library targeting druggable proteins using a novel inducible luciferase based invasion assay. The top 100 hits from the primary screen that showed the strongest decreases in invasion were subjected to confirmation by secondary screening, revealing 24 proteins that are potentially involved in *Toxoplasma* entry into host cells. Interestingly, 6 of the hits appear to affect parasite invasion by modifying host cell actin dynamics, resulting in increased deposition of F-actin at the periphery of the cell. These findings support the emerging notion that host actin dynamics are important for *Toxoplasma* invasion along with identifying several novel host factors that potentially participate in parasite entry.

## Introduction

*Toxoplasma gondii* is an obligatory intracellular parasite classified in the phylum Apicomplexa with other notable pathogens including *Plasmodium*, *Cryptosporidium*, *Eimeria* and *Neospora* spp. *Toxoplasma* is considered as one of the most successful parasites because of its broad host range, usually benign co-existence with the host and propagation by both sexual and asexual means [1]. The parasite has a worldwide distribution in animals and humans. In the United States *Toxoplasma* prevalence was estimated to be 30–40%, resulting in a projected health care burden of at least 5.2 billion dollars per year [2].

*Toxoplasma* infection occurs when a person ingests food or water contaminated with infectious oocysts shed from definitive hosts (felines) or infected meat containing tissue cysts. The parasite differentiates into an actively replicating form termed tachyzoites that disseminates throughout the body, resulting in acute infection [3]. The host immune system responds to tachyzoite replication in an interferon-γ-dependent manner [4] and the parasite differentiates into slowly replicating bradyzoites within tissue cysts that often occupy the nervous system and muscle tissues [5]. The stealthy tissue cysts persist despite a competent host immune system and thus maintain a largely benign life-long infection. However, immunodeficiency from, for example, HIV infection or organ transplantation and anti-rejection drugs can result in bradyzoite differentiation back to actively replicating tachyzoites. Uncontrolled lytic replication in these situations can lead to fatal toxoplasmic encephalitis, pneumonia or myocarditis [6]. Also, transmission of *Toxoplasma* through the congenital route occurs at a frequency of approximately 1 in 1,000 live births in the United States, sometimes resulting in severe birth defects such as blindness, hydrocephaly or cognitive impairment [7].

Since *Toxoplasma* is an obligatory intracellular parasite and host cell invasion is an essential step in its intracellular life, invasion is also a potentially vulnerable stage for intervention. Hence many studies have focused on understanding host cell invasion by *Toxoplasma* with a long-term goal of developing novel therapeutics. *Toxoplasma* uses gliding motility powered by its actomyosin motor system for intercellular transmission and active cell invasion [8], [9]. Host cell invasion by *Toxoplasma* is a multistep process involving the secretion of proteins from apical organelles (micronemes and rhoptries) to form a moving junction (MJ) through which the parasite penetrates into the host cell [10], [11]. The parasite motor system is thought to drive invagination of the host cell plasma membrane, which envelopes the invading parasite and is eventually pinched off leading to the formation of the parasitophorous vacuole (PV). Although many of the key parasite proteins involved in *Toxoplasma* invasion have been characterized, not much is known about the contribution of the host factors to cell invasion. Two recent studies have shown that the host cell is not entirely passive during *Toxoplasma* invasion and that host actin and microtubules contribute to entry [12], [13]. Although these studies suggest involvement of host components in entry, the limited number of such components identified to date restricts our understanding role of the host cell in *Toxoplasma* invasion.

RNA interference (RNAi) mediated gene knockdown is one of the most powerful biological tools available to define protein function [14]. This approach has been successfully used to identify and characterize many novel proteins involved in various aspects of cell biology. Its use in dissecting events in host-pathogen interactions such as microbe entry, intracellular survival, replication and exit has provided new insight into basic cellular processes [15], [16]. Hence to identify additional host factors involved in *Toxoplasma* invasion we performed a high-throughput RNAi screen targeting human druggable proteins. The broad outline of this study was to knockdown the expression of individual host genes, allow parasites to invade and assess for diminished parasite invasion.

However a major requirement for the screen was the availability of a simple, robust and easily quantifiable assay that detects only successfully invaded parasites. Several widely used assays are available to assess parasite invasion, including the β-galactosidase (β-gal) invasion assay [17] and the red-green invasion assay, which is based on differential fluorescence staining [18]. Although the β-gal invasion assay is easy to quantify and has been successfully used to determine total cell-associated parasites, it cannot differentiate between attached and invaded parasites. The red-green assay is considered to be a gold standard to assess *Toxoplasma* attachment and invasion, but it requires many steps, is labor intensive to quantify by microscopy and is prone to inter-experiment variation.

We herein adapt an inducible luciferase-based *Toxoplasma* invasion assay for high-throughput screening and use this assay to newly identify 24 host proteins that are required for efficient *Toxoplasma* cell invasion. Six of identified host proteins showed a nearly identical phenotype of enhanced formation of cortical F-actin upon silencing. Three of these hits were previously implicated in the regulation of actin and cell motility, while the other three have not been linked to actin dynamics and therefore are putative novel regulators of F-actin. Our findings underscore the importance of host actin dynamics in *Toxoplasma* invasion and reveal novel host-derived potential contributors to parasite entry.

## Experimental Procedures

### Parasite Cultures

*Toxoplasma gondii* tachyzoites were maintained by passage through HFF in a humidified incubator at 37°C with 5% CO_2_. The normal growth medium consisted of DMEM supplemented with 10% fetal bovine serum, 10 mM HEPES, 2 mM L-glutamine and 50 µg ml^−1^ penicillin streptomycin. Purification of parasites was performed as described previously [19].

### siRNA Transfection of Host Cells

Host cells were transfected with siRNA using reverse transfection methodology [20]. siRNAs (0.5 pmol/well in 5 µl) from each library plate were transferred to three replica plates using a Biomek FX dual head robot (Beckman), thus each gene was silenced in three replicate wells within different plates screened on the same day. Opaque white Corning CSL3570 plates (Sigma) were used for the luciferase based invasion assay and View plate-384 plates (Perkin Elmer) were used for the red-green high-content microscopy invasion assay. The transfection mix, prepared by 1∶25 dilution of Lipofectamine 2000 (Invitrogen) in OPTI-MEM (Life Technologies), was incubated at RT for 10 min and dispensed onto the plates (5 µl/well) using a multidrop combi liquid dispenser (Thermo Labsystems). The plates were centrifuged briefly and the mixture of siRNA and transfection reagent was incubated at RT for 10 min. HeLa cells were trypsinized, resuspended at 8×10^4^/ml in DMEM (without phenol red) with 10% FBS and added to the plates (40 µl/well i.e., 3.2×10^3^ cells/well) containing siRNAs and transfection reagent. The plates were incubated at 37°C for 72 h to manifest the knockdown of gene expression.

### High-throughput Luciferase Assay

Freshly lysed U-Luc parasites were harvested, filter purified and resuspended in growth medium without phenol red (2×10^7^/ml). The parasites were dispensed (10 µl/well i.e., 2×10^5^ parasites/well) using a multidrop plate dispenser (Thermo Labsystems) into 384-well plates containing siRNA-transfected host cells. The plates were incubated at 37°C for 3 h to allow parasite invasion. After 3 h, plates were removed from the incubator and the liquid in the wells was aspirated using ELx405 plate washer/aspirator (Bio-Tek) leaving approx. 10 µl fluid in each well. CellTiter-Fluor™ (Promega) was dispensed into the plates (5 µl/well), incubated at 37°C for 30 min and the fluorescence (FL) reflecting host cell viability was read using a PHERstar system plate reader (BMG labs). Steadylite Plus™ (Promega) was dispensed onto the plates (10 µl/well), incubated at RT for 10 min and luminescence values reflecting parasite invasion were measured with the PHERstar plate reader. Host cell viability was calculated by normalizing against the negative control (Promega passive lysis buffer) considered 100% viability and the positive control (NT siRNA) considered 0% lysis using the following equation: 100 x (FLtest_well – FLpositive_control)/FLnegative_control – FLpositive_control). Invasion (based on luminescence) was normalized for host cell viability as follows. When percent invasion was positive, the normalized value was the product of percent invasion and percent viability, expressed as percent. When percent invasion was negative, the normalized value was the ratio of percent invasion and percent viability, expressed as percent.

### High-content Microscopy Invasion Assay

Freshly lysed RH-YFP parasites were harvested, filter purified and resuspended in growth medium without phenol red (2×10^7^/ml). The parasites were then dispensed (10 µl/well) using a multidrop system (Thermo Labsystems) into 384-well plates containing siRNA-transfected host cells. The plates were incubated at 37°C for 40 min to allow parasite invasion. Plates were removed from the incubator and washed three times with PBS and fixed, blocked and stained with monoclonal antibody 11–132 (Argene) against SAG1 without permeabilization. After 1 h, plates were washed and Alexa Fluor-594 conjugated goat anti-mouse (Molecular Probes) secondary antibody and DAPI (1 µg/ml; Sigma) were added. After 1 h, plates were washed three times and imaged (nine images per well, 20X S Plan Fluor) with an ImageXpress Micro High-Content Imaging System (Molecular Devices). Red (extracellular parasites), green (total parasites) and blue (host cell nuclei) fluorescent objects of user-defined size and threshold value were enumerated using Molecular devices software MetaXpress, a high-content image acquisition and analysis software (Molecular Devices).

### Data and Statistical Analysis

Primary hits from the screen where established by those with >3SD from the mean invasion values of the non-targeting siRNA samples and >90% host cell viability after gene silencing. A one-sample *t*-test was used to identify hits from the secondary screens that differed significantly (*p*<0.05) from the non-targeting siRNA control.

### Quantitative Reverse Transcriptase-PCR

Total RNA purified from HeLa cells was transcribed into cDNA using the superscript First-Strand synthesis system (Invitrogen) according to the manufacturer’s protocol. qRT-PCR was performed in a 25 µl reaction mixture containing 2X SYBR Green PCR master mix (Stratagene), 10 µM gene specific primer and 0.1 µg of cDNA. Target genes were amplified using an Mx3000P thermal cycler (Stratagene). Melting curve analysis was used to confirm a lack of primer dimers or genomic DNA contamination of reagents. The relative gene expression levels were determined using the 2^−ΔΔCT^ method. GAPDH was used as the control to normalize expression values in cells transfected with either target siRNA or non-targeting siRNA. Primers used in qRT-PCR are listed in supplementary Table S1.

### Immunoblotting

HeLa cell lysates were heated at 100°C for 5 min in SDS-PAGE sample buffer with 2% 2-mercaptoethanol and resolved on 10% or 12.5% polyacrylamide gels. Proteins from the gel were transferred to polyvinylidene fluoride (PVDF) membranes using a semidry transfer apparatus (Bio-Rad) at 16 V for 30 min. After blocking with 10% (w/v) skim milk powder in PBS, membranes were treated with primary antibodies rabbit-TWF2 (Santa Cruz Biotech), rabbit-PHPT1 (Santa Cruz Biotech) or rabbit-MAPK7 (Cell Signaling) for 1 h. Membranes were washed and incubated with secondary antibodies, goat-anti rabbit IgG conjugated to horseradish peroxidase (Jackson Immuno Research Laboratories). After washing, membranes were treated with SuperSignal West Pico chemiluminescent substrate (Pierce Chemical) and exposed to X-ray film.

### Immunofluorescence

Immunofluorescence staining of F-actin in HeLa cells was performed in 8-well chamber slides as described previously [21]. Briefly, slides containing siRNA transfected HeLa cells were washed three times with PBS and fixed at RT with 4% formaldehyde plus 0.025% glutaraldehyde for 20 min. Fixed slides were permeabilized with 0.1% Triton X-100 for 10 min and blocked with 10% FBS for 30 min. After washing briefly, rhodamine-phalloidin (Molecular Probes) diluted in 1% FBS/1% normal goat serum (NGS) was added onto the slides. After 1 h, slides were washed three times and mounted using Mowiol (Sigma-Aldrich). Slides were viewed using an Axio observer Z1 (Zeiss) inverted wide field fluorescent microscope, and digital images were captured using an AxioCam MRm (Zeiss) charge-coupled device camera.

## Results

### Optimization of an RNAi Screen to Assess Toxoplasma Invasion

**Figure 1:**
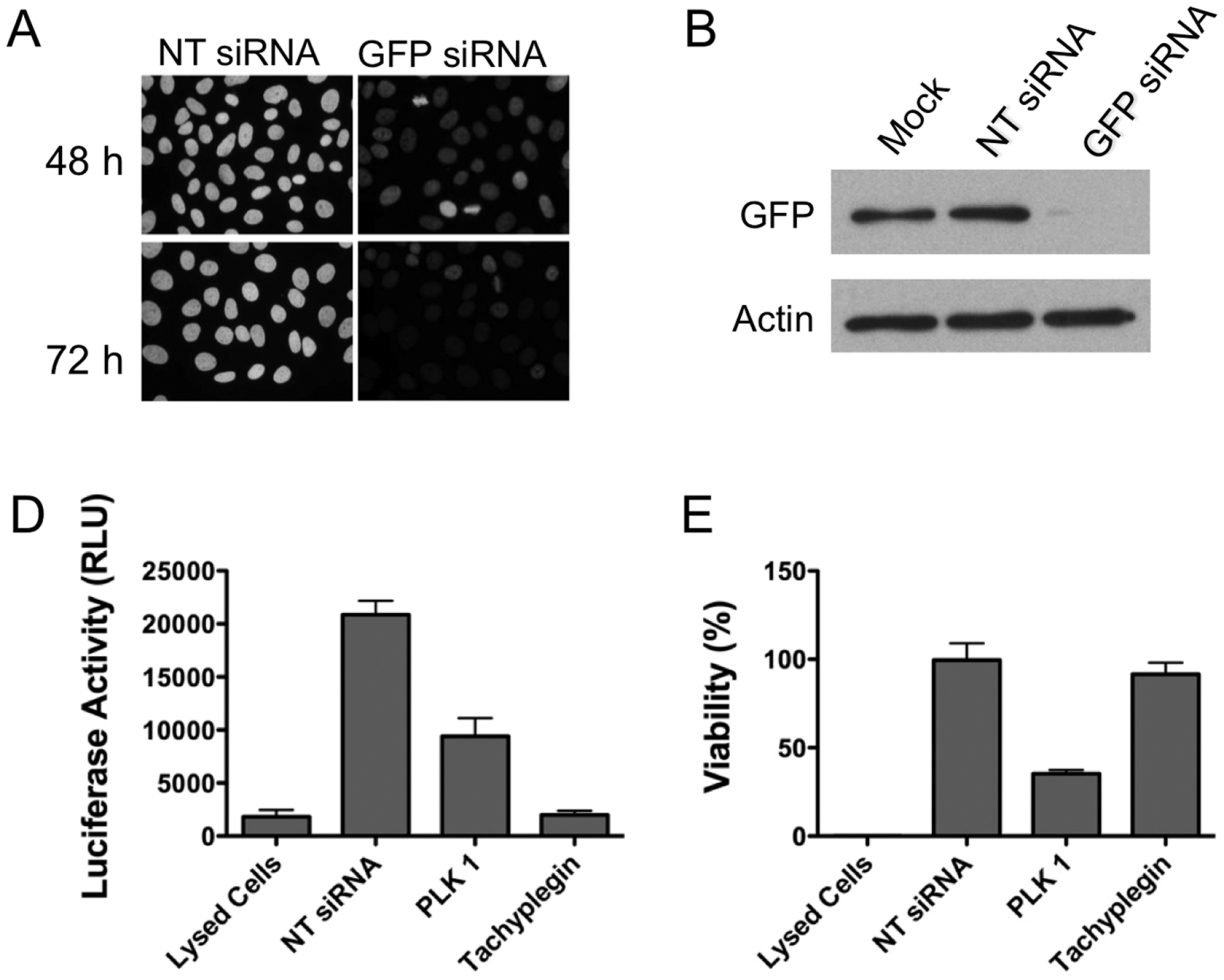
Optimization of target protein knockdown and *Toxoplasma* invasion assay for high-throughput RNAi screen. A. HeLa-GFP cells were transfected with non-targeting (NT) siRNA or siRNA targeting GFP by reverse transfection and examined by immunofluorescence 48 h and 72 h post-transfection. B. HeLa-GFP cells were mock-transfected or transfected with non-targeting siRNA or siRNA against GFP and analyzed by immunoblotting 72 h post-tranfection using anti-GFP or anti-actin antibodies. C. HeLa cells were reverse-transfected with either non-targeting siRNA (lysed cells, NTsiRNA or tachyplegin) or PLK1 in 384-wells. 48 h post-transfection, a set of HeLa cells transfected with NT siRNA were treated with passive lysis buffer to lyse cells. 72 h post-transfection, freshly harvested U-Luc parasites untreated or treated with tachyplegin (chemical inhibitor of invasion) were added onto host cells and incubated at 37°C for 3 h. Steadylite Plus™ was added to the plate and luciferase activity was measured using PHERstar system. *n* = 3 independent experiments each with triplicate samples. Error bars, SEM. D. Host cell viability was assessed by adding CellTiter-Fluor™ and the values obtained with cells transfected with NT siRNA only were set at 100%. *n* = 3 independent experiments each with triplicate samples. Error bars, SEM.

Due to their ease of handling and suitability to plate-based assays, small interfering RNAs (siRNAs) are frequently used for high-throughput gene silencing in highly transfectable cells such as HeLa cells. To optimize the approach and determine the earliest time point where we see efficient target gene knockdown, we made use of HeLa cells stably expressing histone linked to GFP (HeLa-GFP). HeLa-GFP cells were transfected with either non-targeting siRNA (NT siRNA) or siRNA targeting GFP and analyzed by immunofluorescence and immunoblotting. In this and all subsequent experiments, we used a mixture of four siRNAs for each target gene to ensure efficient gene silencing. GFP levels were partially reduced 48 h post-transfection and more substantially diminished 72 h post-transfection (Fig. 1A, 1B). Quantitative reverse transcription PCR (qRT-PCR) showed that the GFP mRNA was reduced by 92.2% 72 h post-transfection. Accordingly, 72 h post-transfection was selected as a suitable time for assessing parasite invasion.

To establish a protocol appropriate for high-throughput identification of host factors involved in *Toxoplasma* invasion, we made use of UPRT-luciferase (U-Luc) reporter parasites in which the firefly luciferase reporter gene is placed under the control of the UPRT promoter [22]. Since the UPRT promoter is induced only after the parasite gains entry into host cells (attached parasites do not up-regulate luciferase), enzymatic activity of luciferase can be conveniently used to quantify parasite invasion. To validate the assay, we reverse-transfected host cells with NT siRNA or siRNAs targeting polo like kinase 1 (PLK1), which is required for cell viability and used here as a proxy for a host gene required for cell invasion [23]. One set of wells containing NT siRNA transfected HeLa cells was lysed with detergent (Lysed Cells) to measure the basal expression of luciferase. In a parallel set of wells transfeced with NT siRNA, U-Luc parasites were allowed to invade HeLa cells for 3 h prior to cell lysis and measurement of induced luciferase expression (NT siRNA). As shown in Fig. 1C, parasite invasion resulted in an ∼12-fold increase in luciferase activity, consistent with previous findings [22]. siRNA silencing of PLK1 reduced invasion by ∼50% according to luciferase activity. Also, treatment with the invasion inhibitory compound tachyplegin [24] substantially reduced luciferase activity as an indicator of invasion. Assessment of cell viability showed that tachyplegin was non-toxic and that silencing of PLK1 reduced cell viability, as expected (Fig. 1D). Together, the above findings establish the effectiveness of siRNA gene silencing under the conditions employed and the suitability of the inducible luciferase assay for assessing invasion in a high-throughput amenable platform.

### Primary Screen of a Library Targeting Human Druggable Proteins

**Figure 2:**
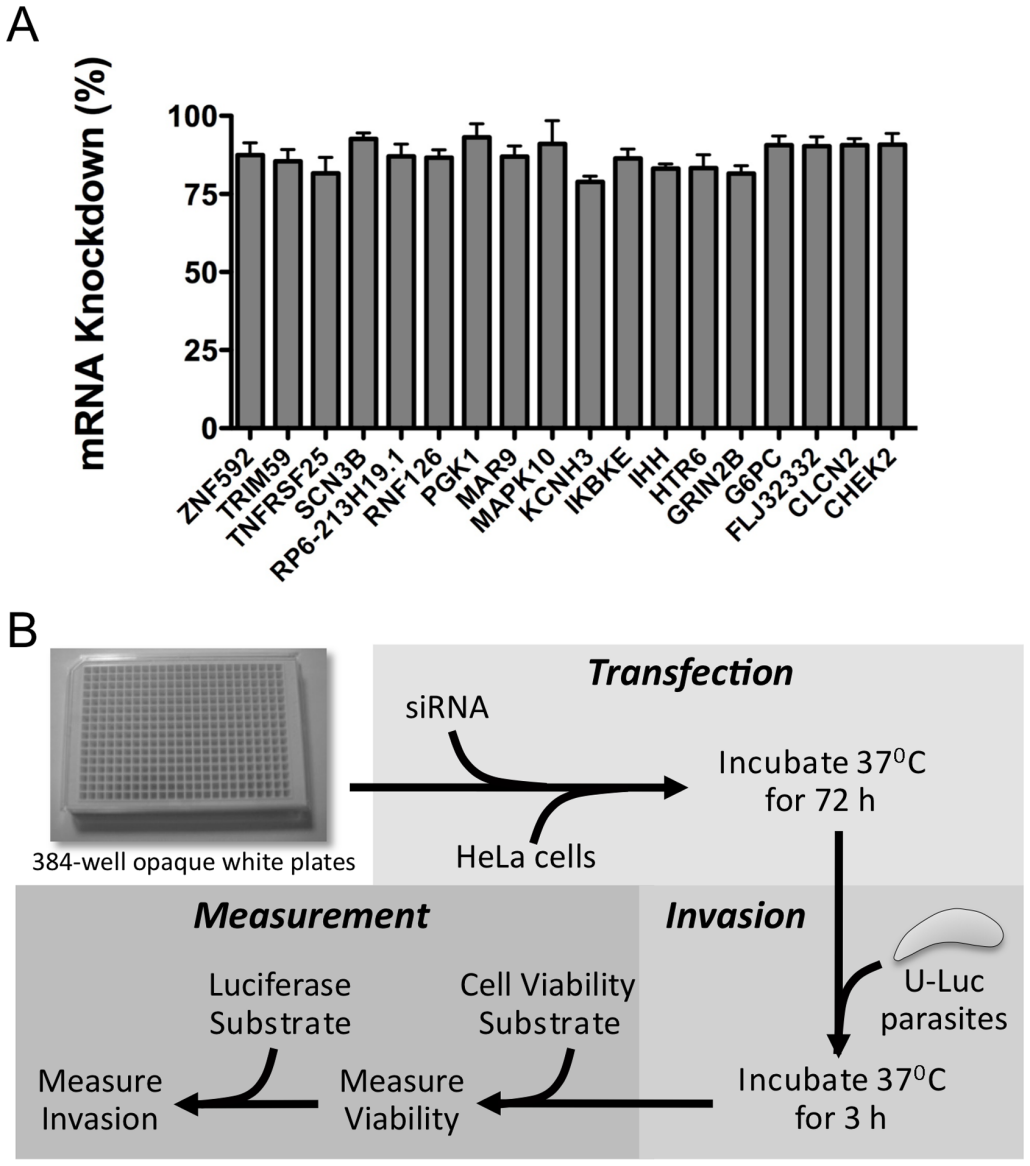
Confirmation of gene silencing in a screening format and overview of the screening procedure. A. Eighteen target genes represented in the library were silenced by siRNA transfection of HeLa cells in 96 well plates. The percent decrease in mRNA levels of target genes was determined using qRT-PCR 72 h post-transfection. *n* = 2 independent experiments, each with triplicate samples. Error bars, SEM. B. Schematic flowchart showing the siRNA screening strategy for identification of host factors playing a role in *Toxoplasma* invasion. The strategy consisted of three main steps of transfection, invasion and measurement. Host cell viability was assessed using CellTiter-Fluor™, which measures protease conversion of a quenched membrane permeable peptide substrate (glycine-phenylalanine-7-Amino-4-trifluormethylcoumarin). Parasite invasion was assessed using the luciferin-based reagent Steadylite Plus™, which measures the inducible luciferase activity of U-luc parasites.

To identify host factors potentially involved in parasite invasion we performed an siRNA screen of a human druggable targets (Thermo Scientific). The library consisted of Smartpool siRNAs, which are mixtures of four siRNAs per target gene that are designed for minimal off-target effects. The library contained siRNAs targeting a total of 2,742 genes, distributed in eleven 384-well plates. To further show the efficacy of siRNAs under screening conditions, we targeted for silencing 18 host genes represented in the library and analyzed mRNA levels by qRT-PCR. The results showed that target gene transcript levels were decreased by at >75% (Fig. 2A).

**Figure 3:**
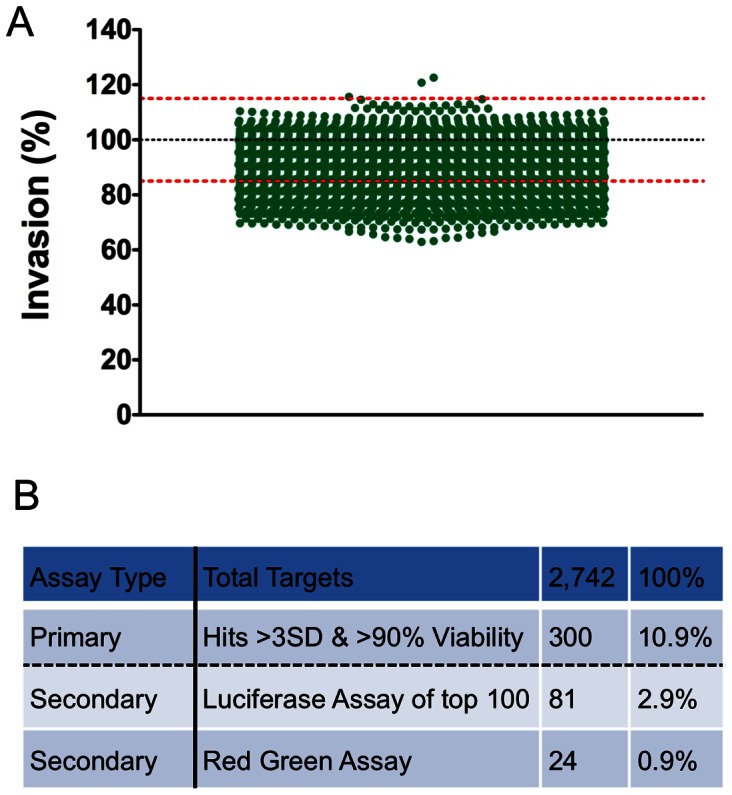
Results of primary screen of an siRNA library targeting human druggable proteins to identify host factors involved in *Toxoplasma* invasion. A. 2,742 genes were screened in triplicate replica plates using U-Luc parasites. The values obtained with NT siRNA controls were set at 100% invasion and the values for target genes were calculated relative to the NT siRNA. The black dotted line represents the mean of the non-targeting siRNA control and the red-dotted lines indicate the 3 standard deviations (SD) boundaries of the mean. Each green dot represents the mean percentage invasion from triplicate samples for one target gene. Only those that showed >90% host cell viability after silencing are shown. B. Table summarizing overall screen. A total of 2,742 genes were subjected to primary screen. Of these 300 genes showed more than >3SD inhibition or enhancement of invasion and >90% host cell viability. The top 100 genes showing inhibition were subjected to secondary screen by luciferase assay and 81 of these were thereby validated. When these genes were subjected to high-content red-green assay, 24 of these showed significant inhibition of parasite invasion.

The protocol for the screen is summarized in Fig. 2B. Briefly, host cells were reverse-transfected with siRNAs in triplicate (i.e., three replica plates) and cultured for 3 days to allow gene silencing before inoculation with freshly harvested U-Luc parasites for 3 hr. Host cell viability and invasion were measured by sequential addition of enzymatic substrate reagents. Mean raw values were converted to percentages of viability and invasion using the non-targeting siRNA negative control values set at 100%. The mean standard deviation of invasion across all test samples was 2.1%, indicating low well-to-well variation amongst the three replicate wells for each sample (Table S2). Percentage invasion was normalized for cell viability. To establish a stringent cut off that eliminates false positives due to loss of cell viability from essential genes, target genes were considered hits if gene silencing inhibited or enhanced invasion by >3 standard deviations (SD) of the negative control (888 genes) *and* host cell viability was >90% of the negative control (1,627 genes). Three hundred hits (10.9%; Table S3) of 2,742 genes screened met this dual cutoff and were therefore considered putative host contributors to *Toxoplasma* invasion (Fig. 3). Silencing 298 of the putative hits resulted in diminished *Toxoplasma* invasion whereas silencing 2 of the hits appeared to enhance invasion. Since we were particularly interested in host components that might contribute to *Toxoplasma* invasion rather than restrict entry, we focus on the former group for follow up analysis.

### Validation of Primary Screen Hits by Luciferase and Red-green Invasion Assays

**Figure 4:**
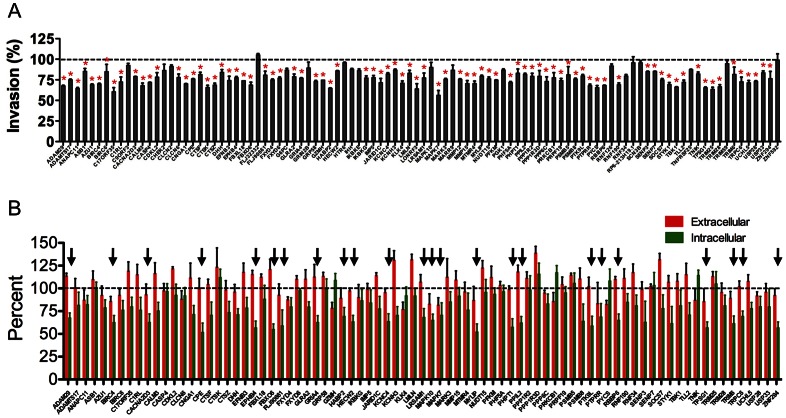
Validation of the primary screen hits with secondary luciferase-based invasion and red-green invasion assays. A. The top 100 hits of primary screen with at least 90% host cell viability were subjected to rescreening with the luciferase invasion assay. The values obtained with non-targeting siRNA controls were set at 100% invasion. A total of 81 genes (red asterisks) showed significant decrease (*p*<0.05, one sample *t*-test) in parasite invasion compared to NTsiRNA controls. *n* = 3 independent experiments each with duplicate samples. Error bars, SEM. B. 81 genes that were validated by secondary luciferase assay were subjected to secondary screen using high-content microscopy based red-green assay. A total of 24 genes (arrows) showed significant reduction (*p*<0.05, one sample *t*-test) in the percentage of invaded parasites in comparison to NT siRNA control. *n* = 4 independent experiments each with triplicate samples. Error bars, SEM.

To validate the findings from the primary screen, we selected the top 100 hits of the 298 that met the initial cutoff for inhibition of *Toxoplasma* invasion and retested them with freshly purchased siRNAs. Retesting these siRNAs in the luciferase-based invasion assay confirmed that 81 of the top 100 genes significantly (*p*<0.05, one sample *t*-test) reduced parasite invasion compared to NT siRNA controls (Fig. 4A). These 81 hits represent 2.9% of the original library. To further investigate a possible role in *Toxoplasma* invasion we adapted the red-green invasion assay, which is considered a gold standard in the field, to a high-content microscopy format. The results showed that silencing 24 of the 81 genes showed decreased invasion compared to NT siRNA control (Fig. 4B). These 24 validated host factors, comprising 0.9% of the original library, represented five functional categories including proteases (6 hits), ubiquitin ligases (8), kinases/phosphatases (4), ion channels (4) and “other” proteins (2) (Table 1). Possible reasons for the attrition of hits between the luciferase assay and red-green assay are provided in the discussion.

**Table 1:** Categorization of the validated hits.

Category	Gene ID	Gene Symbol	Gene Name	UniProtKB	Subcellular Location	Molecular Function	Biological Process
**Proteases**	25823	TPSG1	Tryptase γ	Q9NRR2	Plasma membrane	Serine type endopeptidase	Proteolysis
	4321	MMP12	Matrix metallopeptidase 12	P39900	Extracellular matrix secreted	Calcium ion binding, Zinc ion binding, Metalloendopeptidase activity	Proteolysis
	3026	HABP2	Hyaluronan-binding protein	HABP2	Secreted into extracellular space	Glycosaminoglycan binding, serine type endopeptidase activity	Cell adhesion, proteolysis
	11086	ADAM29	ADAM metallopeptidase domain 29	Q9UKF5	Membrane type I, single pass membrane protein	Metalloendopeptidase activity, zinc ion binding	Proteolysis, spermatogenesis
	1363	CPE	Carboxypeptidase E	P16780	Cytoplasmic secretory vesicle, peripheral membrane protein, secreted	Cell adhesion molecule binding, metallocarboxy pepetidaes activity, zinc ion binding	Proteolysis, protein modification process, Protein localization in membrane
	146547	FLJ90661	Serine protease 36	Q5K4E3	Secreted into extracellular space, extracellular matrix	Serine type endopeptidase activity	Proteolysis
**Ubiquitinases**	26046	ZNF294	Listerin E3 ubiquitin protein ligase 1	O94822	Cytoplasm	Ligase, Zinc ion binding	Ubl conjugation pathway
	57520	HECW2	HECT, C2 and WW domain containing E3 ubiquitin ligase 2	Q9P2P5	Cytoplasm	Ubiquitin protein ligase activity	Ubl conjugation pathway
	5930	RBBP6	Retinoblastoma binding protein 6	Q7Z6E9	Nucleus, Cytoplasm	Nucleic acid binding, protein binding, ubiquitin protein ligase activity, zinc ion biding	Protein ubiquitination involved in ubiquitin dependent catabolic process
	29116	MYLIP	Myosin regulatory light chain interacting protein	Q8WY64	Cytoskeleton, extrinsic to membrane	Cytoskeletal protein binding, ubiquitin protein ligase activity, zinc ion binding	Ubl conjugation pathway
	331	BIRC4	Baculoviral IAP repeat containing 4	P98170	Cytoplasm	Cysteine type endopepetidase inhibitor activity, ubiquitin protein ligase activity	Anti-apoptosis, Wnt receptor signaling pathway
	26270	FBXO6	F-box only protein 6	Q9NRD1	Cytoplasm	Carbohydrate binding, ubiquitin protein ligase	Ubl conjugation pathway
	90678	LRSAM1	Leucine –rich repeat and sterile alpha motif containing protein 1	Q6UWE0	Cytoplasm	Ubiquitin protein ligase	Ubiquitin dependent endocytosis
	117854	TRIM6	Tripartite motif containing 6	Q9C030	Cytoplasm	Zinc ion binding	Protein trimerization
**Kinases/** **Phosphatases**	29085	PHPT1	Phosphohistidine phosphatase	Q9NRX4	Cytoplasm	Protein phosphatase	Regulation of actin cytoskeleton reorganization
	5598	MAPK7	Mitogen activated protein kinase 7	Q13164	Cytoplasm, Nucleus	Serine/threonine protein kinase	Cell cycle, Differentiation
	5801	PTPRR	Protein tyrosine phosphatase, receptor type, R	Q15256	Cell membrane, single pass type 1 membrane protein	Protein phosphatase, receptor, hydrolase	In utero embryonic development
	2050	EPHB4	EPH receptor B4	P54760	Cell membrane, transmembrane protein	Tyrosine protein kinase	Cell adhesion, migration
**Ion channels**	2893	GRIA4	Glutamate receptor, ionotrophic, AMPA4	P48058	Cell membrane, multi-pass membrane protein	Extracellular glutamate gated ion channel activity	Ion transport
	3749	KCNC4	K^+^ voltage gated channel, shaw related subfamily, member 4	Q03721	Membrane, multi-pass membrane protein	Voltage gated potassium channel	Ion transport
	55799	CACNA2D3	Ca^2+^ channel, voltage dependent, α2/δ subunit 3	Q8IZS8	Membrane, type 1 single pass membrane prorein	Voltage gated calcium channel	Ion transport
	7225	TRPC6	Transient receptor protein 6	Q9Y210	Membrane, multi pass protein	Calcium channel	Ion transport
**Other**	11344	PTK9L	Twinfilin, actin binding protein homolog 2	Q61BS0	Cytoplasm, cytoskeleton	Actin monomer binding, protein kinase C binding, phosphatidylinositol-4,5-bisphosphate binding	Barbed-end actin filament capping, sequestering actin monomers
	23759	PPIL2	Peptidylprolyl isomerase (cyclophilin)-like 2	Q13356	Cytoplasm, Golgi lumen	Peptidyl-prolyl ci-trans isomerase activity	Leukocyte migration, blood coagulation, protein folding

### Host Factors that Affect Parasite Invasion by Modifying Actin Dynamics

**Figure 5:**
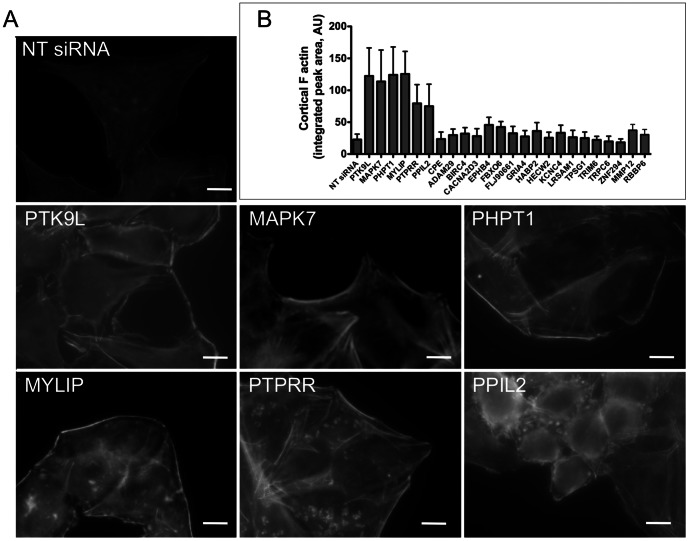
Validated host factors that affect actin dynamics. A. Host cells transfected with NT siRNA or test siRNAs were fixed 72 h post-transfection and stained for F-actin using rhodamine-phalloidin. The images for NT siRNA and test siRNAs were captured with same exposure time. B. Quantification of F-actin at the periphery of the host cell 72 h post-transfection with control (NT siRNA) or test siRNAs. The results are based on three independent experiments each measuring the intensity and thickness of cortical F-actin in 10 cells at four uniformly spaced sites using line-scan analysis with standardized parameters (see also Fig. S2). Error bars, SEM.

Cell invasion by a variety of intracellular microbes is dependent upon the dynamic rearrangement of actin at the site of entry. Recent studies have also implicated host actin dynamics as contributing to the entry of *Toxoplasma* into target cells [12]. These actin rearrangements are influenced by a parasite protein (toxofilin), which locally regulates host actin filament disassembly and turnover [25]. Hence, we examined the list of 24 validated hits to identify proteins that are known to regulate the actin cytoskeleton. Three of the hits - twinfilin 2 (PTK9L), phosphohistidine phosphatase 1 (PHPT1) and mitogen activated protein kinase 7 (MAPK7) – have been previously shown to affect actin dynamics by promoting the formation of F-actin microfilaments [26]–[28], [28,29]. To confirm that we observe a similar effect when these genes are knocked down and also to determine if any of the other 21 genes also affect actin dynamics, we examined the status of F-actin in host cells when these genes are silenced. We transfected host cells with the siRNAs and fixed and stained the cells for F-actin using rhodamine-phalloidin 72 h post-transfection. The results confirmed that cells with knockdown of PTK9L, PHPT1 or MAPK7 showed greater deposition of F-actin near the edge of the cell (Fig. 5A). Quantification of cortical F-actin based on line scan analysis (Fig. 5B and Fig. S1) validated a significant increase in F-actin resulting from target gene silencing. Interestingly, three additional genes - myosin light chain interacting protein (MYLIP), protein tyrosine phosphatase receptor R (PTPRR) and peptidylprolyl isomerase 2 (PPIL2) - also showed a similar phenotype with an apparent fortification of the cortical actin cytoskeleton (Fig. 5A and 5B). None of the remaining 18 validated hits showed consistent changes in cortical F-actin upon gene silencing (Fig. 5B and S2). Together our findings reveal that the silencing of 6 (26%) out of 24 of the validated hits resulted in a similar F-actin enhancing phenotype that is associated with decreased cell invasion by *Toxoplasma*.

**Figure 6:**
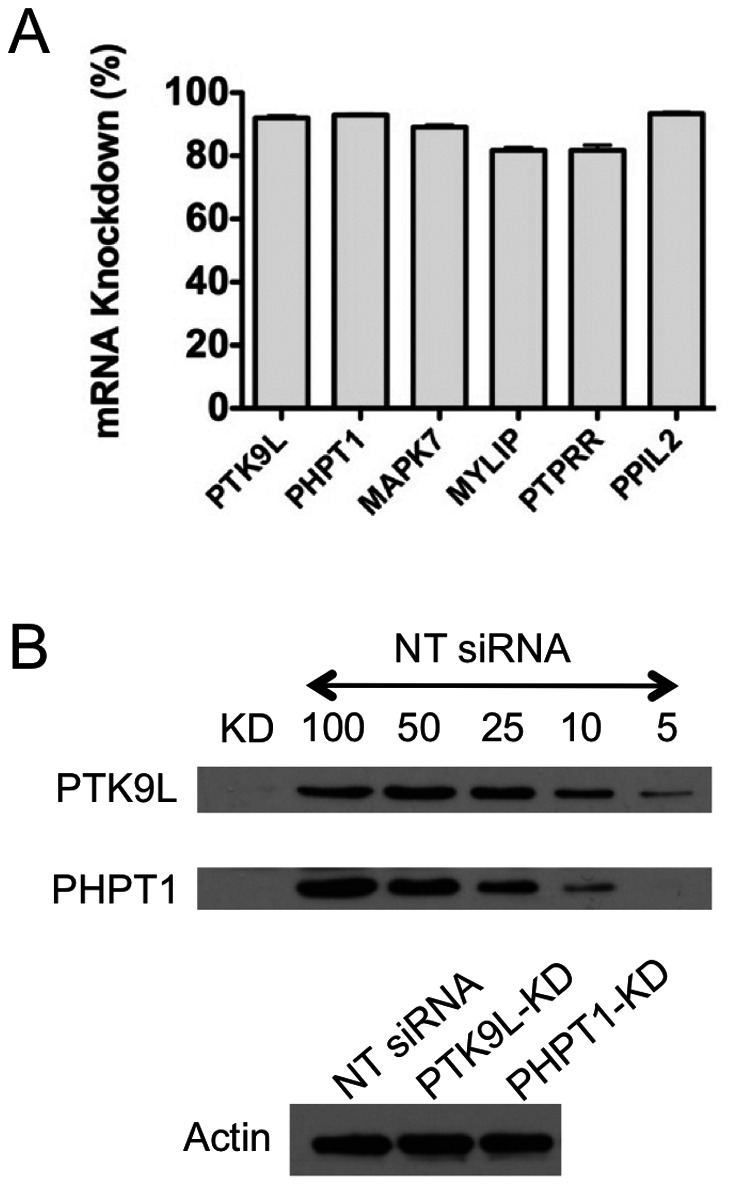
Validation of target gene knockdown at mRNA and protein levels. A. The percent decrease in mRNA levels of target genes transfected with gene-specific siRNA were determined using q-RT PCR 72 h post-transfection. *n* = 2 independent experiments, each in triplicates. Error bars, SEM. B. Immunoblot analysis of host cells transfected with gene-specific siRNA (PTK9L or PHPT1) 72 h post-transfection using anti-PTK9L, anti-PHPT1 and anti-actin (loading control) antibodies. A loading series of the NT siRNA transfected sample (100%, 50%, etc) was included to estimate the percentage knockdown.

To confirm the extent of knockdown at the mRNA level for genes (PTK9L, PHPT1, MAPK7, MYLIP, PTPRR and PPIL2) that showed involvement in actin dynamics we performed qRT-PCR analysis of host cells transfected with specific siRNA. The results showed an 82–95% decrease in the target gene transcript levels 72 h post-transfection (Fig. 6A). We also assessed knockdown of target gene expression at the protein level for two genes (PTK9L and PHPT1) by immunoblotting and the results confirmed a 90–95% knockdown (Fig. 6B). The efficient disruption of mRNA and protein expression for these products is consistent with the observed effects on cortical F-actin dynamics.

## Discussion

As an obligatory intracellular parasite, *Toxoplasma gondii* invasion of host cells is essential for its propagation. Whereas parasite factors involved in cell invasion have been extensively identified and characterized, little attention has been focused on the roles of host components in *Toxoplasma* cell invasion. The prevailing view based on a series of elegant studies is that *Toxoplasma* invasion is an active process that is driven by the parasite whereas the host cell is a largely passive entity [30]. While the evidence in favor of parasite active invasion is extensive and well-accepted, more recent reports demonstrating that host actin and tubulin play important roles during invasion suggest more involvement of host components than previously appreciated [12], [13]. An additional recent report indicates that a *Toxoplasma* myosin (MyoA) and adhesin (MIC2) that were previously thought to be essential for active invasion are not absolutely required for parasite entry [31], thus prompting a reconsideration of the established model. Making use of a novel quantitative invasion assay we herein identify additional host proteins that are required for efficient parasite entry into host cells. Interestingly, a subset of these host factors appears to influence parasite invasion by regulating the host actin cytoskeleton.

A key requirement for using screening approaches to investigate parasite invasion is the availability of an easily quantifiable assay that is suitable for microplate-based screens. Herein we used an inducible promoter-based luciferase assay that is simple and adaptable for high-throughput screening. Luciferase expression is up-regulated only after successful invasion [21], thus eliminating the need to wash out uninvaded parasites and avoiding a potential source of inter-well and inter-experiment variation. Also, luciferase detection is compatible with assessment of cell viability using a fluorescent vital dye, thereby permitting the analysis of host cell viability and parasite cell invasion in the same well. It should be noted, however, that the majority of primary hits that reproducibly showed decreased invasion by the luciferase assay failed to show a significant reduction in cell invasion by the red-green invasion assay. It is possible that some of these apparent false positives affect the progression of the parasite cell cycle from early to late G1, which is required for the up-regulation of luciferase [21]. Although this is a limitation of the assay for assessing invasion, further analysis of the hits that do not reduce cell invasion but diminish luciferase might reveal host genes contributing to parasite initiation of replication. We have also noted high sample-to-sample variation in the red-green invasion assay, which might account for the inability to validate with statistical significance some of the weaker hits. Supporting this notion, the red-green secondary invasion assay showed mean coefficients of variation (COV) of 0.059 and 0.095 for extracellular and intracellular parasites, respectively. Compared to the luciferase secondary invasion assay mean COV (0.026), the red-green COV values are significantly higher (2 sample *t*-test: *p = *9.9×10^−9^ and 3.1×10^−15^, respectively). It should also be noted that our screen likely included false negatives, thus host genes in the library that were not amongst the final hits should not be ruled out as potentially influencing to *Toxoplasma* invasion. Future screens using different invasion assays will likely reveal additional host genes involved in *Toxoplasma* entry.

In addition to genes that diminished parasite invasion when knocked down the primary screen revealed two genes, casein kinase 1 delta (CSN1KD) and SCY1 like 1 (SCYL1), which appeared to enhance invasion upon silencing. An enhancement of invasion upon gene silencing suggests that expression of these genes restricts *Toxoplasma* invasion. Additional studies will be required to confirm or rule out their putative role as inhibitors of *Toxoplasma* entry.

For hits that appear to contribute to *Toxoplasma* invasion, upon gene silencing we observed an inhibition of invasion ranging from 25–40% compared to control NT siRNA. That invasion was not reduced more severely upon silencing of host genes is likely due to several factors. First, siRNA-mediated gene silencing reduces but it does not completely eliminate target protein expression. Our qRT-PCR findings indicate 8–22% residual expression amongst the 24 genes tested (Figs. 2A and 6A). Hence, it is possible that residual protein expression is still capable of supporting vestigial parasite cell invasion. Second, parasite invasion might utilize multiple, partially redundant pathways such that suppressing one pathway leads to an incomplete reduction in invasion. Finally, earlier studies suggest that host cell invasion by *Toxoplasma* is an active process that is less reliant on host components compared to other intracellular microbes. That none of the hits from the present screen are crucial for parasite invasion is consistent with the widely accepted active invasion model. Nonetheless, our findings suggest that host components influence *Toxoplasma* cell invasion and that the entry event may involve extensive interplay between the parasite and host.

**Figure 7:**
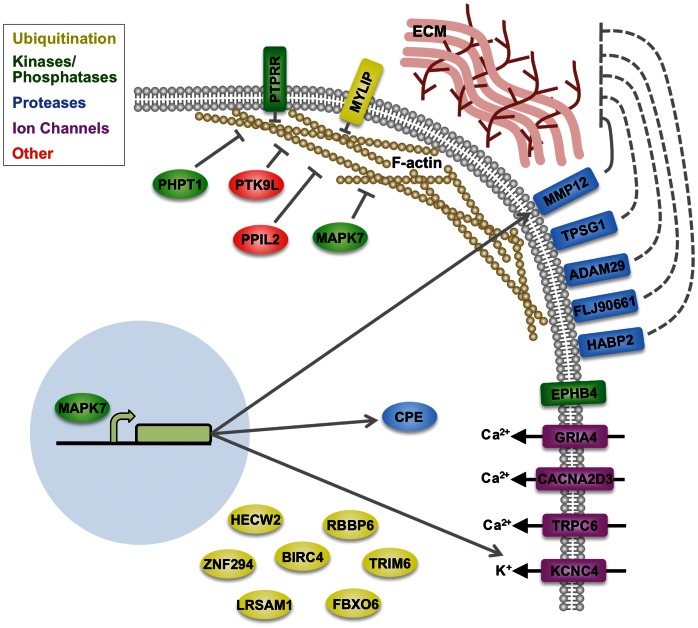
A schematic showing the location of 24 validated host-factors that appear to play roles in host cell entry by *Toxoplasma*. Arrows indicate enhancement of expression or activity whereas T-lines indicate inhibition of expression or activity. Dashed lines indicate hypothetical interactions. Relationships are based on the findings herein or from the literature. See text for additional descriptions.

Of the 24 host factors identified in this screen, six of these are antagonists of the actin cytoskeleton in that down-regulating their expression causes increased development of cortical actin microfilaments. These results are consistent with a previous study implicating host actin dynamics in *Toxoplasma* cell invasion [12], [13]. Of the six proteins, three including PTK9L, PHPT1 and MAPK7 were previously reported to be involved in actin dynamics. PTK9L, also known as twinfilin 2, is a member of the actin depolymerizing factor (ADF)/cofilin family of actin regulating proteins. PTK9L contains two ADF/cofilin domains and inhibits F-actin assembly by sequestering G-actin [32] and by capping the barbed end of F-actin [33]. PHPT1 is a protein histidine phosphatase capable of dephosphorylating phosphohistidine residues on target proteins [34]. Although it not known how PHPT1 regulates actin, targeted knockdown of PHPT1 expression resulted in the redistribution of actin from microspikes and filopodia to cortical microfilaments at the periphery of CL1-5 lung cancer cells [27]. These findings concur with the phenotype we observed upon PHPT1 knockdown in HeLa cervical cancer cells (Fig. 6). MAPK7, also known as ERK5, is a member of the mitogen activated protein kinase family and a central player in cell proliferation, survival, differentiation and migration. MAPK7 promotes dynamic actin cytoskeletal rearrangements for cell migration in part by phosphorylating and inactivating the actin cross-linking protein EPLIN- α [35]. Conversely, down-regulation of MAPK7 is expected to promote EPLIN-αcross-linking, resulting in stabilization of F-actin bundles. Interestingly, by phosphorylating transcription factors, MAPK7 also promotes the expression of several of the other validated hits from our screen including matrix metalloproteinase 12 (MMP12) [36], carboxypeptidase E (CPE) and the potassium voltage-gated channel KCNC4 [37](Fig. 7). Thus, down-regulating MAPK7 could reduce the expression of other host gene products that contribute to *Toxoplasma* cell invasion in an actin-independent manner. It should also be noted that, similar to the actin regulators identified in the screen, other hits could play indirect roles in *Toxoplasma* invasion by regulating the activity of other proteins in the cell.

In addition to verifying deposition of more F-actin near the periphery of the cell when PTK9L, PHPT1 and MAPK7 were knocked down, we also identify three additional proteins - PTPRR, MYLIP and PPIL2 - that negatively regulate cortical F-actin. Although the mechanism of how these proteins contribute to actin dynamics is unclear, the identification of these novel actin-modifying proteins is of significance not only to parasite cell invasion but also to host cell migration and other actin-dependent processes. It should also be noted that, similar to the actin regulators identified in the screen, other hits could play indirect roles in *Toxoplasma* invasion by regulating the activity of other proteins in the cell. Additional studies will be necessary to more deeply understand the contributions proteins identified in the screen make to *Toxoplasma* invasion.

It is interesting to note that more than half of the validated hits are present on the host cell surface where they could act as either receptors for *Toxoplasma* attachment and invasion or be involved in the modification of surface proteins or the extracellular matrix (Fig. 7). Several studies have reported that components of the extracellular matrix including laminin and glycosaminoglycans promote *Toxoplasma* cell invasion [38]–[41]. It is also notable that 5 of the validated hits are surface proteases including MMP12, which contributes to remodeling of the extracellular matrix [42]. We also identified 8 ubiquitin ligases in the screen. Although we do not currently understand how these ubiquitin ligases are involved in parasite invasion especially since their substrates are unknown, one possibility is that they regulate the abundance of surface proteins that interact with the invading parasite. Their precise role in parasite cell invasion awaits further investigation.

## Supporting Information

Figure S1 **Quantification of F-actin thickness at the edge of the cell was done by choosing four equally spaced sites at the periphery of the cell for line-scan analysis.** The area under the peak, above the inflection points on either side was measured using Zeiss Axiovision software.
(TIF)

Figure S2 **Representative images of siRNA knockdown genes that did not consistently affect cortical F-actin.** Host cells transfected with NT siRNA or test siRNAs were fixed 72 h post-transfection and stained for F-actin using rhodamine-phalloidin. All images were captured with the same exposure time.
(TIF)

Table S1 **Primers used in qRT-PCR analysis.** Primer sequences are written from 5′ to 3′.
(DOCX)

Table S2 **Data from the primary screen.** Shown are the gene identification (GENEID) numbers along with the gene names (GENE_SYMBOL). Also shown are luminescence (LUM) values representing cell invasion from the three samples for each gene along with standard deviation (SD). LUM was used to calculate % invasion for each sample (S1, S2, etc) as described in the Experimental Procedures. Fluorescent (FL) values represent host cell viability and were used to calculate % viability.
(XLSX)

Table S3 **List of genes from the primary screen that showed significant (>3SD of the negative control) differences in invasion upon silencing and did not produce substantial host cell death (>90% viability).** Gene identification numbers and names are shown.
(XLSX)
