# Supplementary figures and images for: A Novel High Throughput Invasion Screen Identifies Host Actin Regulators Required for Efficient Cell Entry by Toxoplasma gondii
Source: PLoS One. 2013 May 31;8(5):e64693. doi: 10.1371/journal.pone.0064693 (PMC3669402; doi:10.1371/journal.pone.0064693)

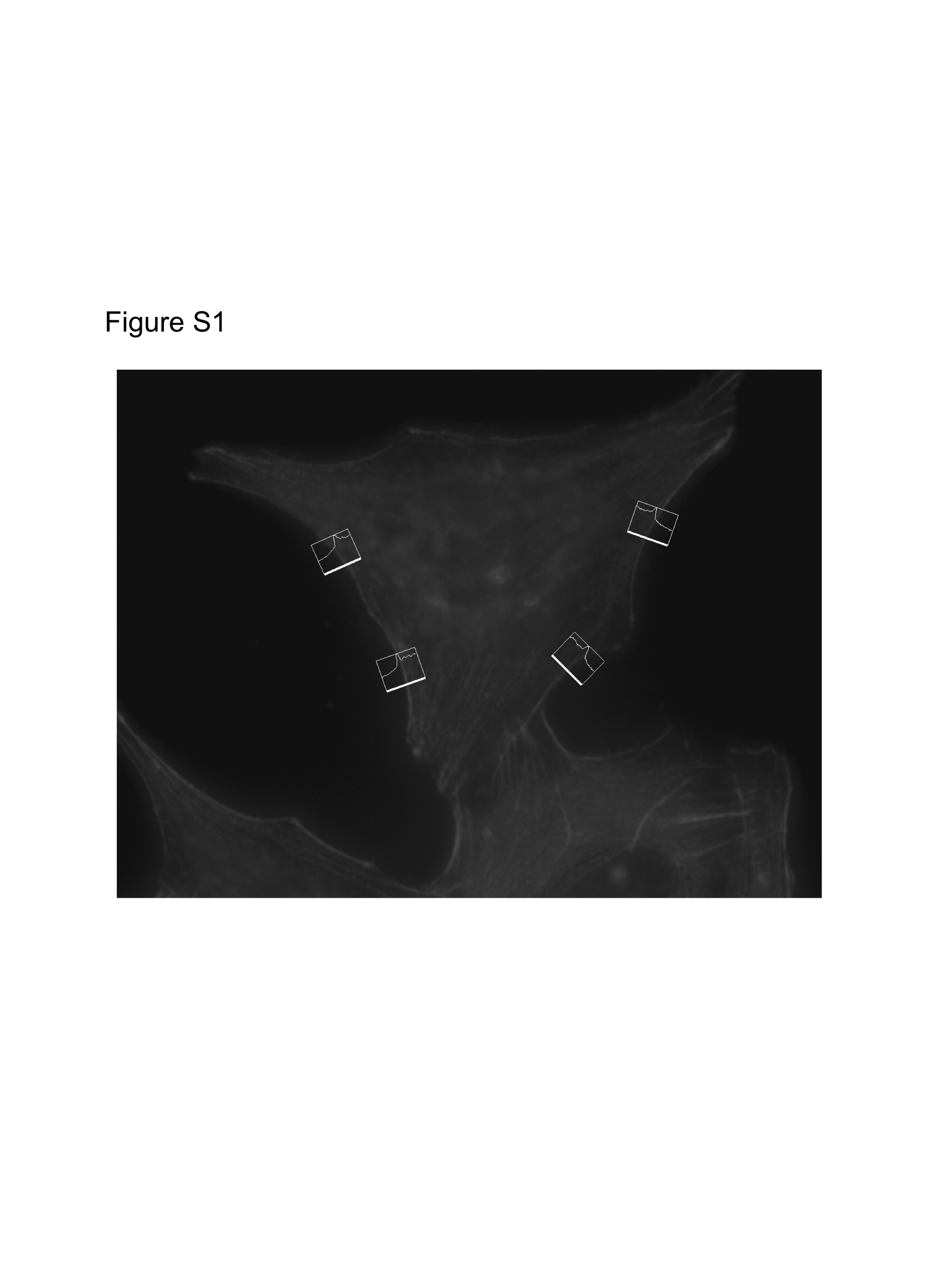

Supplement: Figure S1 — Quantification of F-actin thickness at the edge of the cell was done by choosing four equally spaced sites at the periphery of the cell for line-scan analysis. The area under the peak, above the inflection points on either side was measured using Zeiss Axiovision software. (TIF) [file pone.0064693.s001.tif]

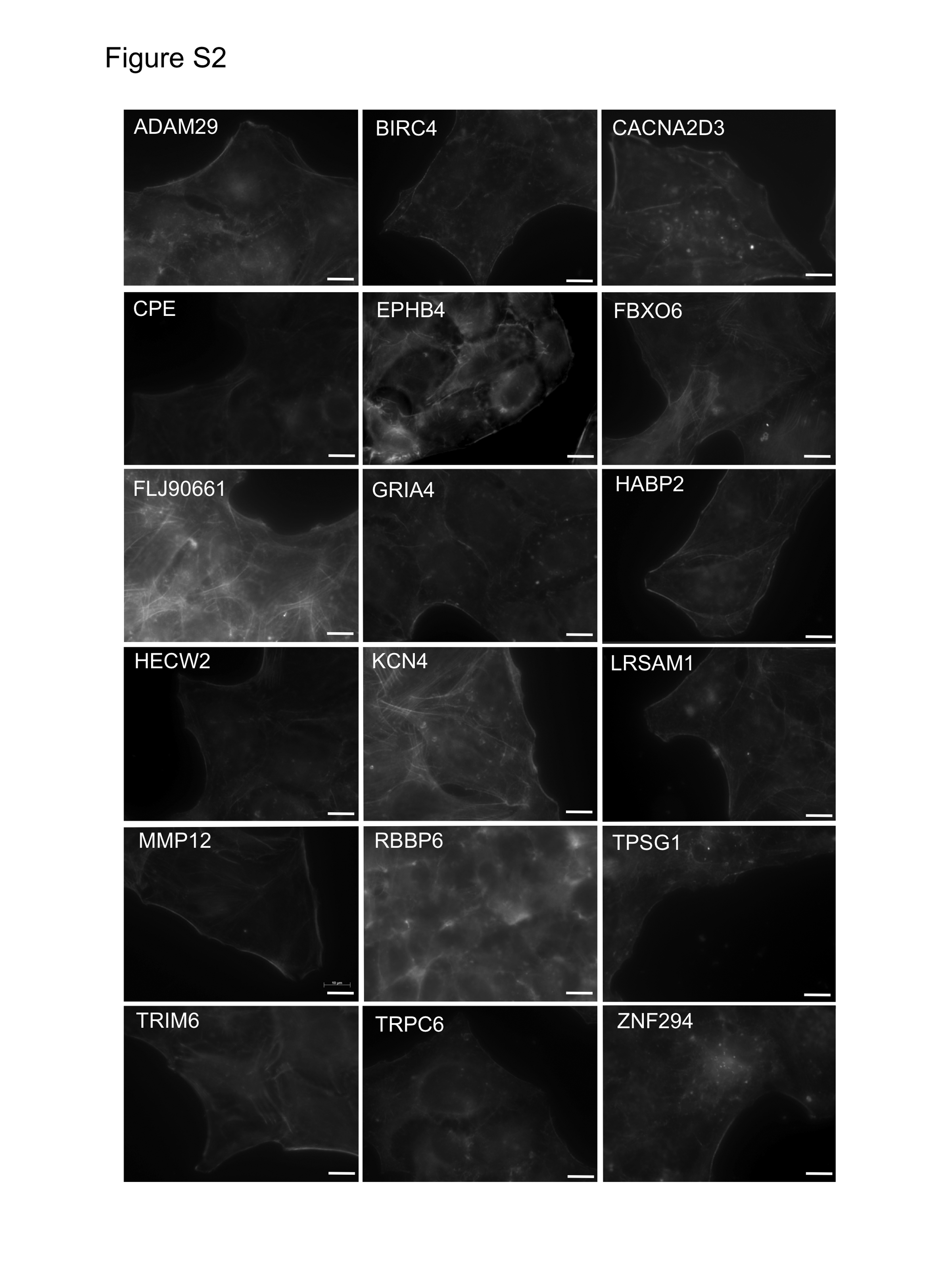

Supplement: Figure S2 — Representative images of siRNA knockdown genes that did not consistently affect cortical F-actin. Host cells transfected with NT siRNA or test siRNAs were fixed 72 h post-transfection and stained for F-actin using rhodamine-phalloidin. All images were captured with the same exposure time. (TIF) [file pone.0064693.s002.tif]
