# Supplementary material for: A Novel High Throughput Invasion Screen Identifies Host Actin Regulators Required for Efficient Cell Entry by Toxoplasma gondii
Source: PLoS One. 2013 May 31;8(5):e64693. doi: 10.1371/journal.pone.0064693 (PMC3669402; doi:10.1371/journal.pone.0064693)
Supplement: Table S1 — Primers used in qRT-PCR analysis. Primer sequences are written from 5′ to 3′. (DOCX) [file pone.0064693.s003.docx]

| 1. TWF2.F | gctgaaggaattctttgccaaggcac |
| --- | --- |
| 2. TWF2.R | GAGCATTCTGTGAGTCGAGGCGGTAG |
| 3. PHPT1.F | tcgctctcattcctgatgtggacatc |
| 4. PHPT1.R | ACTCACAGTCGCAGCCTTGCTTCTGC |
| 5. MAPK7.F | cgctgcctctgtagcggccaagaacc |
| 6. MAPK7.R | TCCGCTTGGCATTGGTCACCACATCG |
| 7. PTPRR.F | ccatcacattacagccagcactgtcc |
| 8. PTPRR.R | CATGTCCAATGTAAGAGATACGTTGG |
| 9. PPIL2.F | aggaacagcacagcgtgcgcacctac |
| 10. PPIL2.R | TGGCCGTGTCCGCATCGCTCTCATAG |
| 11. MYLIP.F | ggcgaaagccaacggcgaggactgcc |
| 12. MYLIP.R | ATGAGGCTCCACGAAGAACTTGACTC |
| 13. GAPDH.F | GGCAAATTCCATGGCACCGTCAAGGC |
| 14. GAPDH.R | TGGTGGTGAAGACGCCAGTGGACTCC |

Table S1. Primers used in qRT-PCR analysis
